# Supplementary material for: Zeb1 facilitates corneal epithelial wound healing by maintaining corneal epithelial cell viability and mobility
Source: Commun Biol. 2023 Apr 20;6:434. doi: 10.1038/s42003-023-04831-0 (PMC10119281; doi:10.1038/s42003-023-04831-0)
Supplement: Supplementary file 2 — Supplementary Materials [file 42003_2023_4831_MOESM2_ESM.pdf]

Supplementary materials

1. Supplementary tables

Supplementary Table S1. Primers used for qPCR detection

Primer name	Sequence (5'—3')	Amplicon (bp)
Hs ACTB LP	GGACTTCGAGCAAGAGATGG	234
Hs ACTB RP	AGCACTGTGTTGGCGTACAG	
Hs CDH1 LP	TTTCCACCAAAGTCACGCTG	162
Hs CDH1 RP	GGCAGCTGATGGGAGGAATA	
Hs FN1 LP	GCAACGATCAGGACACAAGGAC	207
Hs FN1 RP	GGTGAGGCTGCGGTTGGTAA	
Hs ITGA5 LP	CAGATCCTGTCTGCCACTCA	249
Hs ITGA5 RP	GAGGGATCGAATGTCTGAGC	
Hs P15 LP	GGTGAGAGTGGCAGGGTCT	174
Hs P15 RP	CGGGGACTAGTGGAGAAGGT	
Hs P19 LP	ATGCTGCTGGAGGAGGTTC	158
Hs P19 RP	CTGCCAAACATCATGACCTG	
Hs P21 LP	CAGCAGAGGAAGACCATGTG	153
Hs P21 RP	GGCGTTTGGAGTGGTAGAAA	
Hs P27 LP	CCGGCTAACTCTGAGGACAC	221
Hs P27 RP	CTTCTGAGGCCAGGCTTCTT	
Hs GAPDH LP	ATGGGGAAGGTGAAGGTCG	108
Hs GAPDH RP	GGGGTCATTGATGGCAACAATA	
Hs MMP1 LP	ATGCTGAAACCCTGAAGGTG	234
Hs MMP1 RP	CTGCTTGACCCTCAGAGACC	
Hs MMP3 LP	AAGTGGAGGAAAACCCACCT	215
Hs MMP3 RP	TTTCCAGGTCCATCAAAAGG	
Hs MMP11 LP	GCAACCGACAGAAGAGGTTC	135
Hs MMP11 RP	ATACCTTTAGGGCCTCTGCC	
Hs PLAU LP	TCACCACCAAAATGCTGTGT	223
Hs PLAU RP	AGGCCATTCTCTTCCTTGGT	
Hs VIM LP	CGAGGAGAGCAGGATTTCTC	91
Hs VIM RP	GGTATCAACCAGAGGGAGTGA	
Hs ZEB1 LP	CTACAACAACAAGACACTGCTGT	176
Hs ZEB1 RP	TGTTCTTTCAGAGAGGTAAAGCG	
Mm Cdh1 LP	CAGGTCTCCTCATGGCTTTGC	175
Mm Cdh1 RP	CTTCCGAAAAGAAGGCTGTCC	
Mm Fn1 LP	AGGCAGGCTCAGCAAATCGT	157
Mm Fn1 RP	GGCTTCCTCCATAGCAGGTACA	
Mm Gapdh LP	AACGACCCCTTCATTGAC	191
Mm Gapdh RP	TCCACGACATACTCAGCAC	
Mm Mmp2 LP	TGACCTTGACCAGAACACCA	171
Mm Mmp2 RP	AAAGCATCATCCACGGTTTC	
Mm Mmp7 LP	CCCTGTTCTGCTTTGTGTGTCT	177
Mm Mmp7 RP	GCATTTCCTTGAGGTTGTCCAC	
Mm Nfkb LP	GCTTTGCAAACCTGGGAATA	150
Mm Nfkb RP	TCAGGTCCATCTCCTTGGTC	

Mm p15 LP	CCCTGCCACCCTTACCAGA	169
Mm p15 RP	CAGATACCTCGCAATGTCACG	
Mm p16 LP	CCCAACGCCCCGAACT	79
Mm p16 RP	GCAGAAGAGCTGCTACGTGAA	
Mm p21 LP	GTGGCCTTGTCGCTGTCTT	126
Mm p21 RP	GCGCTTGGAGTGATAGAAATCTG	
Mm p27 LP	TTGGGTCTCAGGCAAACTCT	157
Mm p27 RP	TCTGTTCTGTTGGCCCTTTT	
Mm Tnfa LP	CGTCGTAGCAAACCACCAAG	241
Mm Tnfa RP	GGCAGAGAGGAGGTTGACTT	
Mm Vim LP	CGGCTGCGAGAGAAATTGC	124
Mm Vim RP	CCACTTTCCGTTCAAGGTCAAG	
Mm Zeb1 LP	TGGCAAGACAACGTGAAAGA	200
Mm Zeb1 RP	AACTGGGAAAATGCATCTGG	

Supplementary Table S2. Primers used for ChIP-PCR

Primer name	Sequence (5' - 3')	Amplicon (bp)
Mm Nfkb Pro LP	GGCACTTTGCCCAAAGAATA	162
Mm Nfkb Pro RP	ATCATCGCTGTGGATGGAAT	
Mm Tnfa Pro LP	CTACCTGGCCATGACAACCT	231
Mm Tnfa Pro RP	ACTTTCCGAACACCCTTCCT	
Hs TNFR1 Pro LP	CTGAGGTGGGCAGATCATTT	170
Hs TNFR1 Pro RP	CGACCTCCCAGGTTCAAGTA	
Hs CDH1 Pro LP	TAGAGGGTCACCGCGTCTAT	200
Hs CDH1 Pro RP	TCACAGGTGCTTTGCAGTTC	
Hs GAPDH Pro LP	TACTAGCGGTTTTACGGGCG	166
Hs GAPDH Pro RP	TCGAACAGGAGGAGCAGAGAGCGA	
Hs FN1 Pro LP	AAAGAAAGGGAGCGGGATGGG	190
Hs FN1 Pro RP	GGGTGGTGGTAGTGTTTGAGGA	
Hs MMP11 Pro LP	ATGGAACAATCTCGGCTCAC	177
Hs MMP11 Pro RP	AGGTCAGGAGTTGGAGAGCA	
Hs P21 Pro LP	TATTAGCTGGGCATGGTGGT	177
Hs P21 Pro RP	GCAGCCCTGGCTTTTTGTTT	
Hs PLAU Pro LP	ATATCTGGGGACTGCCACTG	170
Hs PLAU Pro RP	CCAACTTGCCTAAGACTGCC	

2. Supplementary figures.

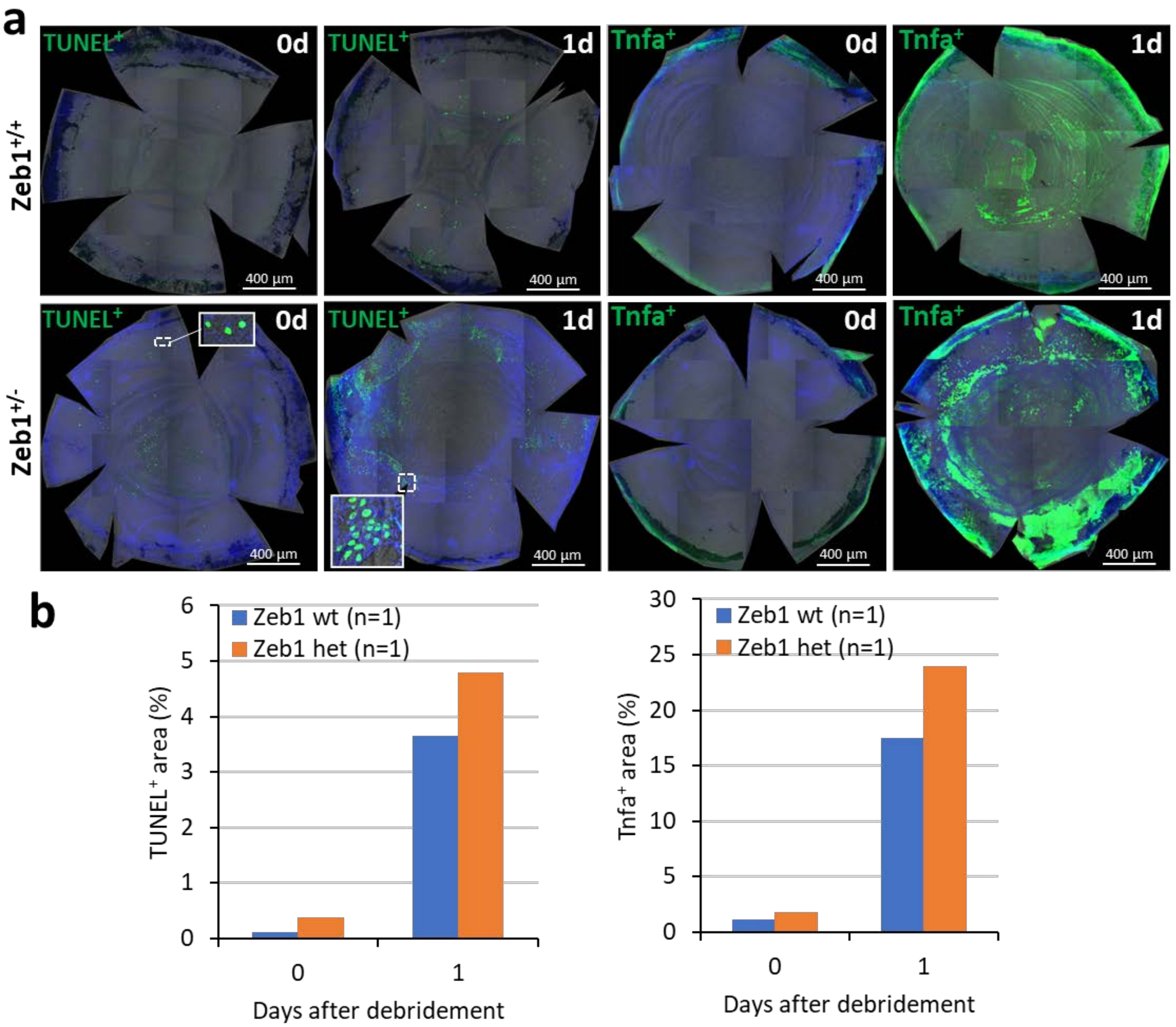

Supplementary Figure S1. Wholemount immunostaining evaluation on corneal apoptotic cell death and Tnfa secretion before and after the debridement. (**a**) To validate the results obtained by immunohistochemistry (IHC) on the corneal cross sections (Fig. 2 and Fig. 3) we conducted a corneal wholemount immunostaining to detect $TUNEL^+$ cell death and $Tnfa^+$ secretion using both $Zeb1^{+/+}$ (wt) and $Zeb1^{+/-}$ (het) corneas collected before (0d) and 1 day (1d) after the mechanical debridement. (**b**) The ratios of either $TUNEL^+$ or $Tnfa^+$ area to the related total corneal area indicated that the mechanical debridement and the monoallelic deletion of Zeb1 mutually increased corneal apoptotic cell death and Tnfa secretion.

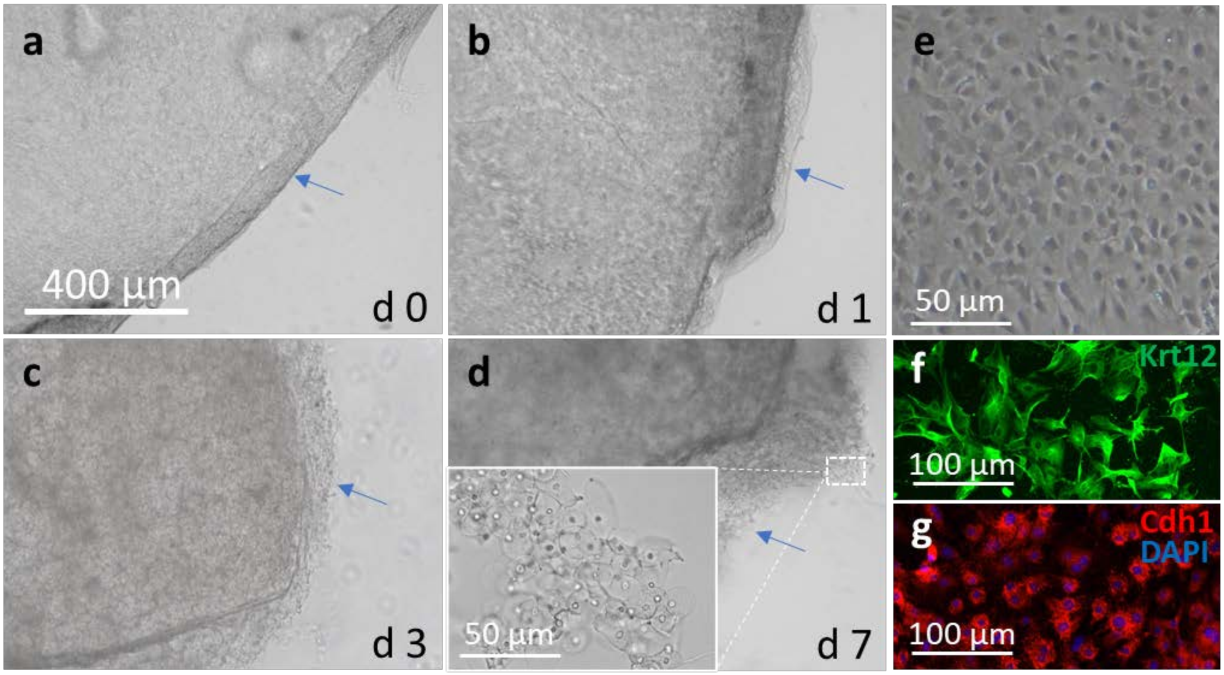

Supplementary Figure S2. Mouse corneal epithelial cell isolation and culture. The isolated mouse cornea is placed upside down on cell culture plate coated with 0.1% gelatin for **(a)** 0, **(b)** 1, **(c)** 3, and **(d)** 7 days. **(e)** The monolayer-cultured corneal epithelial cells and their immuno-fluorescence with **(f)** the corneal epithelial-specific marker Krt12 and (**g**) the common epithelial marker E-cadherin (Cdh1).

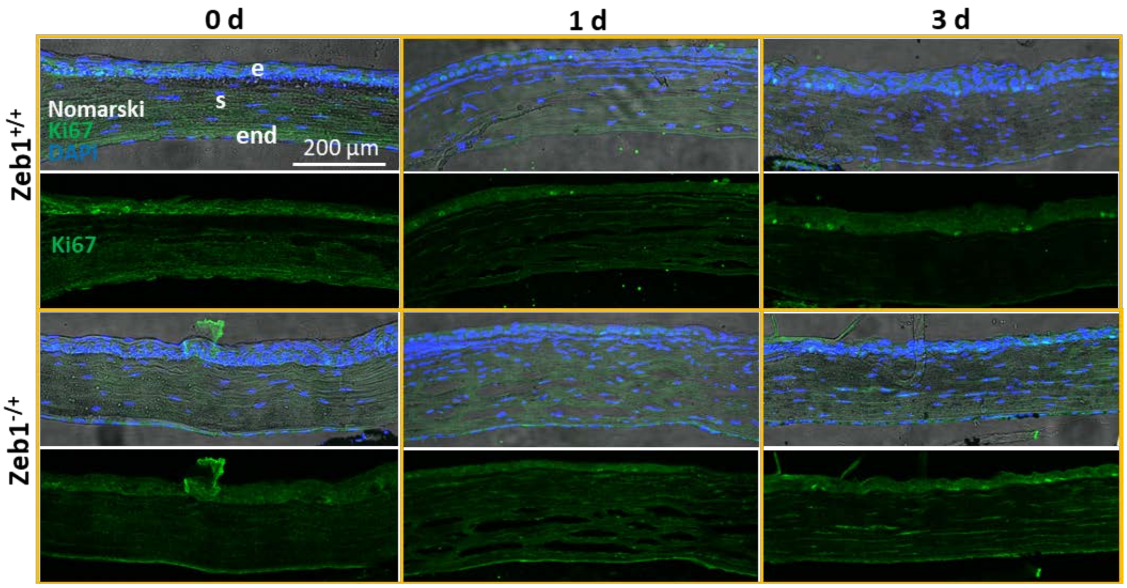

Supplementary Figure S3. The Expression of the proliferative marker Ki67 in the central corneas of both Zeb1+/+ and Zeb1-/+ before (0d) and 1d and 3d after the epithelial debridement. e, epithelium; s, stroma; end, endothelium.

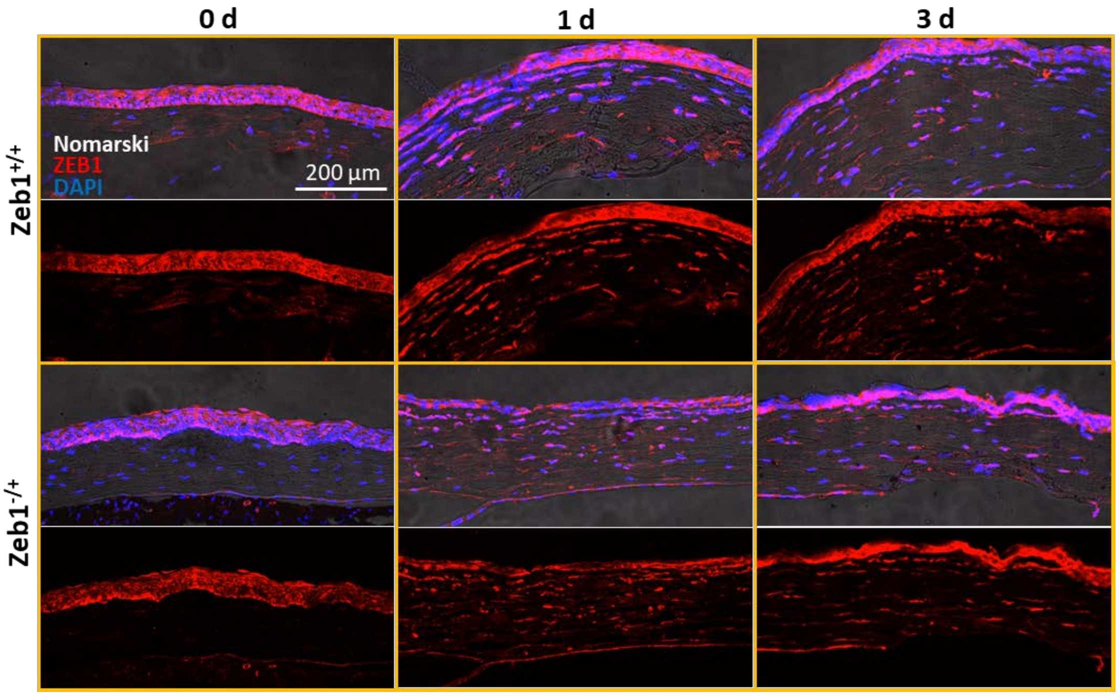

Supplementary Figure S4. The Expression of Zeb1 in the central corneas of both Zeb1+/+ and Zeb1-/+ before (0d) and (1d and 3d) after the epithelial debridement.

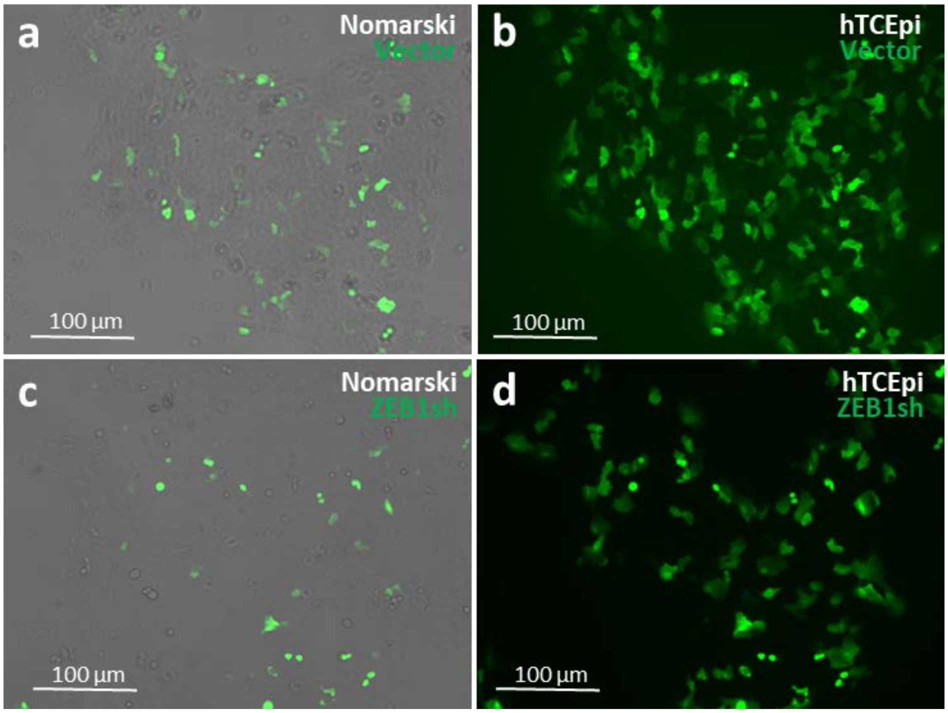

Supplementary Figure S5. hTCEpi cells infected with the vector control (Vect Ctrl) or ZEB1sh lentivirus. Both Vect Crtl and ZEB1sh express GFP, thereby the infection rates were calculated based on the GFP-expressed cells. After 3 passages in culture, (a), (b) the rate of Vect Ctrl cells was retained 80% with the GFP (c), (d) while that of the ZEB1sh was declined to 50%.

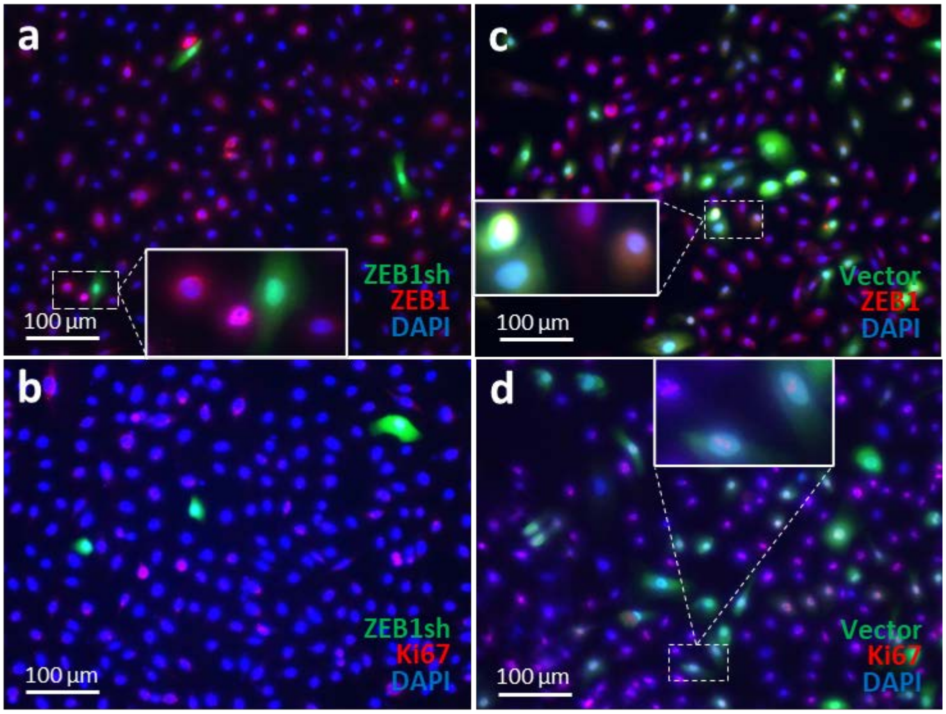

Supplementary Figure S6. hTCEpi cells infected with the vector control (Vect Ctrl) or ZEB1sh lentivirus. After passage 7 times, only 1 – 2% of the hTCEpi cells showed the ZEB1sh GFP and their expression of both (a) ZEB1 and (b) Ki67 detected by the immunofluorescence was significantly lower than the rest cells without ZEB1sh GFP. Meanwhile about 50% of the hTCEpi cells showed the Vect Ctrl GFP and their expression levels of both (c) ZEB1 and (d) Ki67 were similar to the rest cells without ZEB1sh GFP

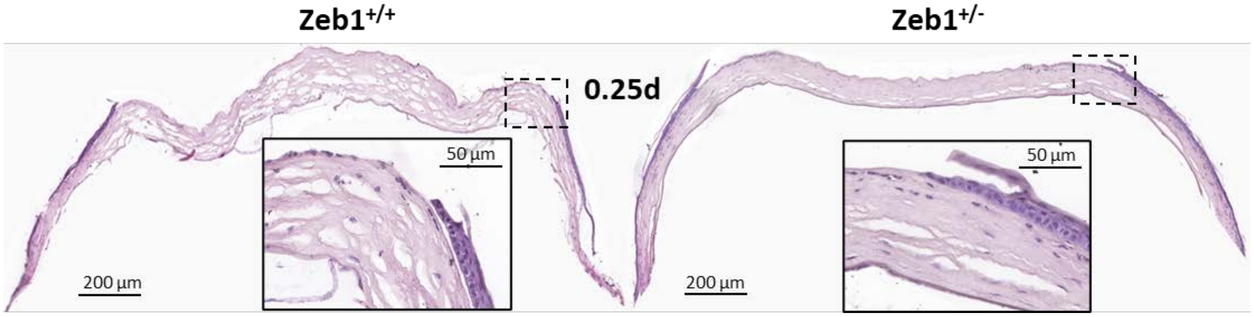

Supplementary Figure S7. H&E histological assessment on the debrided corneas. To clarify whether the central corneal epithelium is smoothly removed by the Alger Brush II under a stereo microscope, representative corneas of both Zeb1$^{+/+}$ and Zeb1$^{+/-}$ mice were collected in 6 hours (0.25d) after the mechanical debridement, and then fixed, paraffin-embedded, sectioned and H&E stained. It appeared the mechanical debridement of the corneal epithelium was done well with few basal cells scattered in the debrided areas.
